# Updated National Diagnostic Reference Levels and Achievable Doses for CT Protocols: A National Survey of Korean Hospitals

**DOI:** 10.3390/tomography8050203

**Published:** 2022-09-29

**Authors:** Sora Nam, Hyemin Park, Soonmu Kwon, Pyong-kon Cho, Yongsu Yoon, Sang-wook Yoon, Jungsu Kim

**Affiliations:** 1 Department of Bio-Convergence Engineering, Korea University Graduate School, Seoul 02841, Korea; 2 Center for Radiological Environment & Health Science, Dongseo University, Busan 47011, Korea; 3 Department of Radiological-Technology, Daegu Health College, Daegu 41453, Korea; 4 Department of Radiologic Science, Daegu Catholic University, Gyeongsan 38430, Korea; 5 Department of Multidisciplinary Radiological Science, The Graduate School of Dongseo University, Busan 47011, Korea; 6 Department of Diagnostic Radiology, CHA Bundang Medical Center, CHA University, Seongnam 13496, Korea

**Keywords:** diagnostic reference levels, national survey, archivable dose

## Abstract

**Background:** In 2021, the Korean government proposed a new CT diagnostic reference level. This study performed a nationwide survey and developed new DRLs and AD for 13 common CT examinations. We compared other countries’ DRLs for CT examinations. **Methods:** This study investigated the CTDI_vol_ and DLP of the 12 types of CT protocols for adults and brain CT protocol for pediatrics. A total of 7829 CT examinations were performed using 225 scanners. We defined the DRLs values in the distribution of radiation exposure levels to determine the nationwide patient dose and distribution status of the dose. **Results:** This study showed that the new Korean national CT DRLs are slightly higher or similar to those of previous surveys and are similar or lower than those of other countries. In some protocols, although the DLP value increased, the CTDI_vol_ decreased; therefore, it can be concluded that the patient’s dose in CT examinations was well managed. **Conclusions:** The new CT DRLs were slightly higher than or similar to that of the previous survey and were evaluated to be similar or lower than CT DRLs of other countries. These DRLs will be used for radiation optimization and effective dose calculation for an individual.

## 1. Introduction

After the computed tomography (CT) was developed in 1973 [1], this technology has rapidly changed. The helical scanning technique and multidetector-row scanning technology were introduced in the late 1980s [2] and 1990s, respectively [3]. Today CT scanning technology is more focused on reducing the radiation dose to the patient using artificial intelligence image reconstruction algorithms. In the clinical field, CT examination is a powerful tool for the diagnosis and management of a patient’s disease. However, CT examination has two different sides: one increases the diagnostic accuracy and the other increases the radiation exposure risk of the population. Americans were more than seven times likelier to be exposed to ionizing radiation for medical procedures in 2006 than in the early 1980s. In the US, 18.3 million cases had undergone CT procedures in 1993; the number steadily increased from 1993 to 2011, accounting for 82.0 million cases in 2016 [4]. 

The International Atomic Energy Agency (IAEA) and World Health Organization (WHO) have recommended the diagnostic reference levels (DRLs) to diagnostic radiology departments for patients in each country and region [5,6,7]. The DRLs were proposed by the International Commission on Radiological Protection (ICRP) publication 73 in 1991 [7]. According to the ICRP Publication 103, one of the principles for optimization of protection during medical exposures is implemented using DRLs [8]. The DRLs has proven to be an effective tool that aids in the optimization of protection during medical exposure of patients for diagnostic and interventional procedures. The DRLs are a supplement to professional judgement and do not provide a dividing line between good and bad medical practice. All individuals who have a role in subjecting a patient to medical exposure should be familiar with DRLs as a tool for the optimization of protection [9]. National and regional DRLs should be revised at regular intervals of 3–5 years or more frequently when substantial changes in technology, new imaging protocols, or improved post-processing of images have occurred [9]. Many countries have established national DRLs for diagnostic radiologic procedures. National DRLs should be compared with regional or local DRLs, and if the DRLs of individual medical institutions are higher than the national DRLs, each center should implement the quality control procedures to keep their DRLs lower than the national DRLs, and the procedures should be established to optimize. The Korean government established the first DRLs of CT examination in 2008; however, the DRLs were established for CT examinations of the head, chest, and abdomen. In 2016, the Korean government established the second national DRLs for CT examinations [10]. In 2021, the Korean government proposed new CT DRLs. We performed a nationwide survey and developed new DRLs of 25% and 75% values and achievable doses (AD) for 12 common CT examinations and pediatric brain CT examinations. Further, we compared other countries’ DRLs for CT examinations. 

## 2. Materials and Methods

This survey complied with the Health Insurance and Portability and Accountability Act and was approved by the institutional review boards prospectively. The patient radiation dose information in CT examinations typically includes both the CT dose index volume (CTDI_vol_) and dose length product (DLP). This survey was investigated for digital imaging and communications in medicine (DICOM) radiation dose structured report information and dose report of DICOM images. We investigated CTDI_vol_ and DLP of the whole examination of 12 adult CT protocols and pediatric brain CT protocols from March to November 2021. Over this period, a total of 7186 adults and 643 pediatric brain CT examinations were performed using 225 CT scanners. In 2021, 2030 CT scanners were installed in Korea, and the survey was performed using 11.08% of the installed scanners. Table 1 shows the installed CT numbers in regional distribution and the surveyed number of CT. We investigated the number of health insurance service through the Korean health insurance big data system from November 2019 to October 2020, and the highest number of cases using 12 adult protocols was selected. Patients were selected by body weight, based on 5–95% of Korean standard body types taken from a Korean body type survey from the Korean National Statistical Office. In the 7th Korean Standard Body Survey 2015, the quintiles value and 95 quartile value of height and weight were 151.2 cm, 179.8 cm, 47.0 kg, and 87.0 kg, respectively, in 15 to 70 year olds [11]. Patients’ data within this range were collected and used the value which displayed in the CT device, and this study did not calculate the DLP separately. BMI was not considered in this study. Table 2 shows average age, weight, and height of CT protocols.

The CT protocol was the most frequent billing CT examination of the national health insurance service. The 12 adult CT protocols included brain CT without contrast, intra-cranial CT angiography, cervical spine CT, lumber spine CT, neck CT with contrast, chest CT with contrast, low-dose screening chest CT, abdomen-pelvis CT with contrast, abdominal 4-phase dynamic CT with contrast, abdomen-pelvis CT without contrast for urinary system, coronary artery CT angiography, and coronary artery CT calcium score. The pediatric brain CT without contrast examinations were stratified into four groups according to patient age. The data collection was performed by two methods: e-mail survey and offline survey, using the CTDI_vol_ and DLP information collection system of DICOM information, including DICOM information and DICOM dose structured report. The surveyed data were analyzed with the SPSS version V18 (IBM, Armonk, NY, USA). We defined the DRLs as the 25th and 75th percentile values in the distribution of radiation exposure levels to determine the nationwide patient dose and distribution status of the dose. We defined the AD as the 50th percentile in the distribution of radiation exposure levels in each CT protocol. This study obtained at least five patient data per CT device in each exam protocol in accordance with the recommendations of ICRP [9]. In addition, all the CT devices that were surveyed in this study passed the CT quality control program conducted by the Korea Centers for Disease Control and Prevention, which included evaluating the accuracy of CTDI_vol_ and DLP. This study was approved by the institutional review boards of Bundang Cha Hospital (approval number CHAMC 2021-03-011-002).

## 3. Results

**Figure 1:**
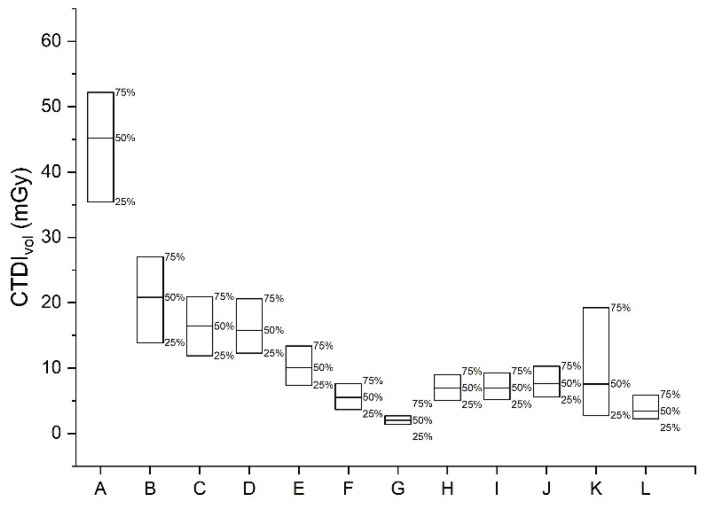
The 25th and 75th percentile DRLs and 50th percentile (achievable dose) CTDI_vol_ values for computed tomography (CT) examinations of the chest, abdomen, and other body parts. A: Brain CT without contrast, B: Intra-cranial CT angiography, C: Cervical spine CT, D: Lumber spine CT, E: Neck CT with contrast, F: Chest CT with contrast, G: Low dose screening chest CT, H: Abdomen-pelvis CT with contrast, I: Abdomen 4 phase dynamic CT with contrast, J: Abdomen-pelvis CT without contrast for urinary system, K: Coronary artery CT angiography, L: Coronary artery calcium score CT.

**Figure 2:**
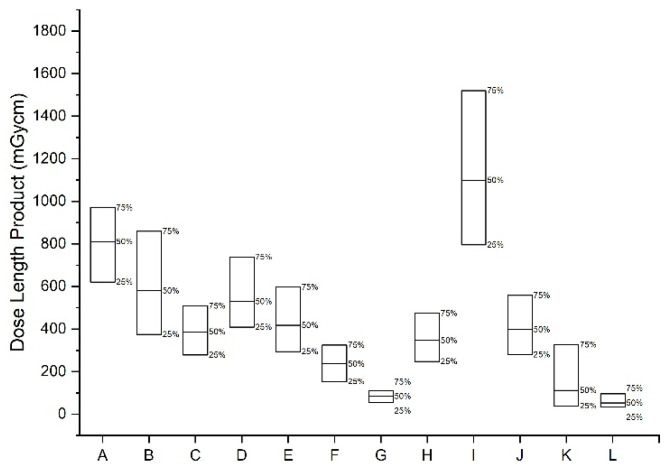
The 25th and 75th percentile DRLs and 50th percentile (achievable dose) DLP values for computed tomography (CT) examinations of the chest, abdomen, and other body parts. A: Brain CT without contrast, B: Intra-cranial CT angiography, C: Cervical spine CT, D: Lumber spine CT, E: Neck CT with contrast, F: Chest CT with contrast, G: Low dose screening chest CT, H: Abdomen-pelvis CT with contrast, I: Abdomen 4 phase dynamic CT with contrast, J: Abdomen-pelvis CT without contrast for urinary system, K: Coronary artery CT angiography, L: Coronary artery calcium score CT.

This survey collected nationwide data from various medical institutions. Figure 1 and Figure 2 show the 25th and 75th percentile DRLs and AD values of CTDI_vol_ and DLP for CT examinations of the brain, chest, abdomen, and other body parts. This survey included 10.58% brain CT without contrast, 10.28% low-dose screening chest CT examinations, 10.80% chest CT examinations with contrast, 10.33% lumber spine CT, and 9.45% abdomen-pelvis CT examinations with contrast. All the examinations were performed using 225 CT scanners. The CT examinations were performed in clinics (3.9%), general hospitals (12.2%), university hospitals (83.7%), and health promotion centers (0.1%).

### 3.1. National DRLs and AD

The DRLs and ADs of CTDI_vol_ and DLP values obtained from this survey are presented in Table 3. Figure 1 shows the 25th and 75th percentile DRLs and AD CTDI_vol_ values for CT examinations. Figure 2 shows the 25th and 75th percentile DRLs and AD DLP values for CT examinations. Table 4 shows the 25th and 75th percentile DRLs and AD values of CTDI_vol_ and DLP for each group of pediatric brain CT without contrast.

### 3.2. Exposure Conditions

For the adult brain CT without contrast, the average tube voltage, tube current time product, and scan length were 122 ± 7.8 kVp, 289.1 ± 100.2 mAs, and 173.2 ± 30.9 mm, respectively. For the pediatric under two years of age brain CT without contrast, the average tube voltage, tube current time product, and scan length were 102.0 ± 9.5 kVp, 204.4 ± 139.7 mAs, and 150.6 ± 17.5 mm, respectively. The 12 adult CT protocols and average tube voltage, tube current time product, and scan length are presented in Table 5.

## 4. Discussion

The use of DRLs has been shown to reduce the overall radiation dose and range of radiation doses received by patients undergoing radiologic procedures. In this study, the new Korean national CT DRLs were developed following the second piece of research conducted in 2016 [10]. The study showed that the new Korean national CT DRLs are slightly higher or similar to those of the second piece of research and are similar or lower than those of other countries. In some protocols, although the DLP value increased, the CTDI_vol_ decreased; therefore, it can be concluded that the patient’s dose in CT examinations was well managed. The main reason for the increase in DLP value is presumed to be the expansion of the scan area because of the introduction of multi-detector CT. 

In the case of an adult brain CT, the CTDI_vol_ and DLP were lowered by 11 mGy and 149 mGy·cm, respectively, in the 2019 survey (third), when compared with the second Korean national CT DRLs [10]. In addition, it was confirmed that it was managed at a low dose, compared to the US, Europe, and Japan. The result, in comparison with other countries, showed both the CTDI_vol_ and DLP values of this study to have relatively lower DRLs than that of the American College of Radiology (ACR) Dose Index Registry (DIR), Japan, European Union (EU), and Canada, and it was similar to Australia. However, the DLP of this study was found to be relatively higher than that of Australia, which means that Korea scans a relatively longer area. The CTDI_vol_ and DLP were found to have increased, compared to the 2016 survey of the intra-cranial CT angiography, neck CT with contrast, cervical spine CT, lumbar spine CT, chest CT with contrast, low dose screening chest CT, and abdomen-pelvis CT without contrast for urinary system. As a result of comparing the DRL for neck CT with contrast with other countries, the CTDI_vol_ of this study was relatively lower compared to ACR DIR and Australia; however, the DLP showed a higher value than the ACR DIR and Australia. This also means that the scan length is longer in the Korean CT examination. 

When comparing the DRLs of cervical spine without contrast material CT, it was confirmed that the DLP result of this study was higher than in the EU or Australia. The lumbar spine CT was also found to have a lower CTDI_vol_ than Australia; however, the DLP value was higher. In the case of chest CT with contrast, compared to the ACR DIR, EU, Australia, and Canada, both CTDI_vol_ and DLP showed lower values in this study. Similarly, the low dose screening chest CT showed lower values for both CTDI_vol_ and DLP, compared to EU, indicating that Korean CT is well managed (Table 6).

In the case of abdomen-pelvis CT with contrast and abdomen 4 phase dynamic CT with contrast, the results of this study and 2017 survey showed similar values. In this study, the coronary artery CT angiography showed a lower dose, compared to the 2017 survey, however, coronary artery calcium score CT showed an increased dose. However, considering all factors, the overall Korean national CT DRLs was found to be lower, compared to other countries. Similarly, in this study, the DRLs value increased slightly compared to the 2017 survey for pediatric brain CT without contrast. Moreover, compared with other countries, it was found to be of a higher value than Australia, lower value than Japan, and a value similar to that of the United Kingdom and Canada (Table 7). However, the period of DRLs survey and the age of the patients in the comparative country do not exactly match, and more detailed research is required in the future. In the case of intra-cranial CT angiography, comparing it with the DRL in other countries is difficult; therefore, an additional study for this protocol should be considered.

In the third survey compared with the second survey, in the case of adult brain CT and intracranial CT angiography, the CTDI_vol_ and the DLP of AD value were decreased by 11.60 mGy, 181.90 mGy·cm, 0.13 mGy, and 31.20 mGy·cm, respectively. In the case of the cervical spine CT, the CTDI_vol_ and DLP of AD value were increased by 1.92 mGy, 53.00 mGy·cm. In the case of the lumbar spine CT, the CTDI_vol_ of AD value was decreased by 0.12 mGy, however, the DLP of AD value was increased by 34.98 mGy·cm, compared with the second survey.

In case of the neck CT with contrast, low dose screening chest CT, abdomen-pelvis CT with contrast, and abdomen 4 phase dynamic CT with contrast, the CTDI_vol_ and DLP of AD value were decreased by 0.97 mGy, 21.71 mGy·cm, 0.15 mGy, 3.09 mGy·cm, 0.85 mGy, 4.31 mGy·cm, 1.75 mGy, 92.60 mGy·cm, respectively, compared with the second survey. 

However, in the case of chest CT with contrast and coronary artery calcium score CT, DLPs were only increased by 9.27 mGy·cm and 268.10 mGy·cm, respectively. In case of the abdomen-pelvis CT without contrast for urinary system, the CTDI_vol_ and DLP of AD value were increased by 1.01 mGy and 64.36 mGy·cm, respectively. In case of coronary artery CT angiography, the CTDI_vol_ and DLP of AD value were decreased by 14.11 mGy and 230.72 mGy·cm, respectively. In most protocols, the AD values of the third survey were decreased, compared to the second survey; however, the increment of AD DLP in cervical spine CT, lumber spine CT, abdomen-pelvis CT without contrast for urinary system, and the coronary artery calcium score CT meant that the scan area became widened. Therefore, in case of these protocols, lowering the target value by lowering the scan range setting will be the way to achieve optimization [10].

The purpose of the exam and using the CT device might be different depending on the size of the medical institutions. In the results of this study, it was confirmed that CTDI_vol_ was rather higher at the university and the general hospital level than at the clinic level. Table 8 shows the comparison of average CTDI_vol_ and DLP for the medical institutions site size group.

In addition, based on the results of this study, the effective dose evaluation with tissue weighting factor will be easily used as a tool for evaluating the patient effective dose in the clinical area [8]. 

## 5. Conclusions

In addition to the DRL of this study, the utilization of advanced dose reduction technologies, such as using the AI, will be a useful way to reduce patients’ dose.

The use of advanced dose reduction technologies, such as the AI, is an excellent technology to reduce patients’ dose. However, most CTs lack this advanced technology. The DRLs of this study, along with advanced technologies, will serve as an important basis reference for reducing patient dose in most existing CTs.

In conclusion, this survey provides the 3rd set of data for national CT DRLs for 12 adult CT protocols and a pediatric head CT protocol in Korea in 2021. The new Korean national CT DRLs was evaluated to be slightly increased or similar to that of the second survey and was evaluated to be similar or lower than the CT DRLs of the other countries. However, for some protocols, the DRL for DLP, compared to the DRL for CTDI_vol_, was relatively high, compared to the results in other countries. Therefore, since the CT scan in Korea has a relatively longer scan length, compared to other countries, adjustment for this should be considered. The DRLs derived from this study can be used as data for quality control of radiologic examinations in the medical field.

## Figures and Tables

**Table 1:** Regional distribution number of installed CT and surveyed number of CT in 2021.

Region Name	Number of Installed CT	Surveyed Number of CT
Seoul and Gyeonggi area	898	93
Kang won area	73	8
Gyeongsang area	574	79
Jeolla and Jeju area	283	26
Chungcheong area	202	19
Total	2030	225

**Table 2:** Patients’ average age, average weight, and average high of adult CT protocols.

CT Protocol Name	Ages (Years)	Hight (cm)	Weight (kg)
Brain CT without contrast	58.74 ± 18.14	164.80 ± 11.76	66.90 ± 14.04
Intra-cranial CT angiography	59.29 ± 14.33	164.06 ± 8.94	64.02 ± 12.13
Cervical spine CT	53.73 ± 16.90	166.86 ± 9.60	66.69 ± 12.40
Lumber spine CT	58.92 ± 17.26	165.90 ± 54.48	66.12 ± 12.95
Neck CT with contrast	53.82 ± 18.09	165.46 ± 8.50	64.88 ± 11.26
Chest CT with contrast	62.32 ± 16.21	164.59 ± 8.75	64.91 ± 37.67
Low dose screening chest CT	57.63 ± 15.13	165.07 ± 11.64	66.99 ± 35.90
Abdomen-pelvis CT with contrast	56.72 ± 18.91	163.75 ± 20.79	68.62 ± 75.56
Abdomen 4 phase dynamic CT with contrast	58.48 ± 14.16	175.27 ± 87.10	74.41 ± 83.01
Abdomen-pelvis CT without contrast for urinary system	59.66 ± 18.08	165.47 ± 18.84	68.00 ± 44.18
Coronary artery CT angiography	58.66 ± 15.82	165.96 ± 8.77	66.99 ± 11.21
Coronary artery calcium score CT	55.30 ± 13.31	166.77 ± 8.96	65.45 ± 11.08

**Table 3:** 25th and 75th percentile DRLs and Ads of CTDI_vol_ and DLP values for adult CT examinations.

Protocols	CTDI_vol_ (mGy)			DLP (mGy·cm)		
	25th	AD	75th	25th	AD	75th
Brain CT without contrast	35.4	45.2	52.2	621.0	811.0	969.8
Intra-cranial CT angiography	13.9	20.8	27.0	374.9	579.8	858.9
Cervical spine CT	11.9	16.4	20.9	278.0	387.0	508.7
Lumber spine CT	12.3	15.8	20.6	409.6	529.6	738.5
Neck CT with contrast	7.4	10.0	13.4	294.4	417.3	597.1
Chest CT with contrast	3.6	5.5	7.6	154.0	236.7	324.2
Low dose screening chest CT	1.4	2.0	2.7	54.0	84.4	109.5
Abdomen-pelvis CT with contrast	5.0	6.9	8.9	247.9	346.6	473.7
Abdomen 4 phase dynamic CT with contrast	5.2	7.0	9.3	796.0	1099.7	1521.8
Abdomen-pelvis CT without contrast for urinary system	5.6	7.6	10.3	280.0	398.2	558.5
Coronary artery CT angiography	2.8	7.6	19.2	38.5	112.2	326.9
Coronary artery calcium score CT	2.2	3.4	5.9	34.7	53.5	95.7

**Table 4:** 25th and 75th percentile DRLs and Ads of CTDI_vol_ and DLP values of age groups for pediatric brain CT without contrast examinations.

Protocols	Age Group	CTDI_vol_ (mGy)			DLP (mGy·cm)		
		25th	AD	75th	25th	AD	75th
Pediatric brain CT without contrast	under 2 years	15.5	19.7	23.5	236.9	323.8	429.3
	more than 2 years—under 5 years	20.3	24.6	31.4	341.4	429.3	585.0
	more than 5 years—under 10 years	23.0	30.0	38.5	422.0	534.0	756.0
	more than 10 years—under 15 years	30.0	45.0	51.5	581.4	826.0	967.2

**Table 5:** The scan parameter for the 12 adult CT protocols.

CT Protocol Name	Average Tube Voltage (kVp)	Average Tube Current Time Product (mAs)	Average Scan Length (mm)
Brain CT without contrast	122.0 ± 7.8	289.1 ± 100.2	173.2 ± 30.9
Intra-cranial CT angiography	113.2 ± 12.7	171.9 ± 98.3	255.5 ± 102.2
Cervical spine CT	119.6 ± 10.6	216.5 ± 274.7	245.1 ± 64.3
Lumber spine CT	119.7 ± 7.7	298.6 ± 368.4	327.8 ± 149.4
Neck CT with contrast	114.8 ± 10.8	161.9 ± 143.6	303.2 ± 47.6
Chest CT with contrast	115.1 ± 10.0	113.9 ± 120.4	380.8 ± 18.0
Low dose screening chest CT	117.0 ± 7.5	45.0 ± 45.3	370.8 ± 62.5
Abdomen-pelvis CT with contrast	110.9 ± 11.2	183.7 ± 305.7	436.1 ± 91.2
Abdomen 4 phase dynamic CT with contrast	109.8 ± 11.9	194.1 ± 196.0	373.8 ± 102.4
Abdomen-pelvis CT without contrast for urinary system	112.1 ± 11.3	169.3 ± 179.7	428.1 ± 251.6
Coronary artery CT angiography	114.7 ± 10.7	139.9 ± 139.9	169.4 ± 75.8
Coronary artery calcium score CT	118.3 ± 7.0	79.4 ± 78.1	175.6 ± 84.9

**Table 6:** Comparison of 75th % DRLs between other countries and the current survey of adult CT protocols.

Body Part, Examination, and Parameter	ACR DIR (2017) [12]	NCRP (2012) [13]	Japan (2020) [14]	EU (2021) [15]	Ireland (2010) [16]	Australia (2020) [17]	Canada (2018) [18]	Netherlands (2012) [19]	Saudi Arabia (2022) [20]	Nigeria (2005–2019) [21]	Korea (2016) [10]	This Study (2021)
CT of head and brain without contrast material												
CTDI_vol_	57	75	77	48	66/58	52	79		33.1	67	63	52.2
DLP	1011		1350	1386	940	880	1302		655.74	1410	1119	969.8
CT of intra-cranial angiography with contrast material												
CTDI_vol_											22	27.0
DLP											836	858.9
CT of neck with contrast material												
CTDI_vol_	20					17					14	13.4
DLP	572					450					442	597.1
CT of cervical spine without contrast material												
CTDI_vol_				17		23					18	20.9
DLP				495		470					434	508.7
CT of cervical spine with contrast material												
CTDI_vol_	28				19							
DLP	602				420							
CT of lumbar spine without contrast material												
CTDI_vol_						26					18	20.6
DLP						670					601	738.5
CT of chest without contrast material												
CTDI_vol_	15	21	13	9	9		14		31.7			
DLP	545		510	364	390		521		637.01			
CT of chest with contrast material												
CTDI_vol_	16	21				10	14				7	87.6
DLP	596					390	521				297	324.2
CT of low dose screening Chest												
CTDI_vol_											3	2.7
DLP											101	109.5
CT of chest pulmonary arteries with contrast material												
CTDI_vol_	18			8	13			10				
DLP	557			628	430			350				
CT of abdomen and pelvis without contrast material												
CTDI_vol_	20	25	20	9	12		18	15	32.17			
DLP	1004		880	874	600		874	700	645.93			
CT of abdomen and pelvis with contrast material												
CTDI_vol_	19	25			12	13	18	15			10	8.9
DLP	995				600	600	874	700			472	473.7
CT of abdomen and pelvis dynamic with contrast material (4 phase)												
CTDI_vol_			17	9							10	9.3
DLP			2100	1273							1511	1521.8
CT of abdomen, pelvis, and kidney without contrast material												
CTDI_vol_	18			8		13					9	10.3
DLP	877			480		600					460	558.5
CT of Coronary Angiography												
CTDI_vol_			66	25							30	19.2
DLP			1300	459							447	326.9
CT of Calcium score												
CTDI_vol_				4							5	5.9
DLP				81							77	95.7

CTDI_vol_ unit: mGy, DLP unit: mGy·cm

**Table 7:** Comparison of 75th % DRLs between other countries and the current survey of pediatric brain CT protocols.

Age	US (2021) [22]		Australian (2020) [17]		Japan (2020) [14]		Canada (2018) [18]		Tunisia (2017–2019) [23]		Korea (2016) [10]		This Study (2021)	
	CTDI_vol_	DLP	CTDI_vol_	DLP	CTDI_vol_	DLP	CTDI_vol_	DLP	CTDI_vol_	DLP	CTDI_vol_	DLP	CTDI_vol_	DLP
0	23	344	30	470	30	480	37	578	26	384	20	298	24	429
1	27	440			40	660			38	664				
2														
3	31	518					51	843			24	404	21	585
4														
5			35	600					51	873				
6					55	850					30	494	38	756
7	55	910												
8							52	888						
9														
10									51	978				
11					60	1000					63	1087	51	967
12														
13														
14														
15														
16														
17														
18														

CTDIvol unit: mGy, DLP unit: mGy·cm.

**Table 8:** Comparison of average CTDI_vol_ and DLP between medical institutions site size group.

Examination	Clinic		Hospital		University Hospital	
	CTDI_vol_	DLP	CTDI_vol_	DLP	CTDI_vol_	DLP
CT of head and brain without contrast material	39.27	638.35	58.76	998.4	45.4	831.09
CT of intra-cranial angiography with contrast material	13.26	508.02	67.08	1114.03	23.39	653.15
CT of cervical spine without contrast material	17.4	369.01	20.62	467.56	19.45	413.31
CT of Lumbar spine without contrast material	17.9	565.6	41.86	698.35	16.7	576.08
CT of low dose screening Chest	1.34	54.04	2.52	103.3	2.21	85.9
CT of chest with contrast material	4.76	206.08	9.85	353.72	6.6	250.27
CT of Coronary Angiography	11.77	199.14	12.78	212.68	13.65	229.54
CT of Calcium score	3.18	47.12	4.74	73.6	4.71	74.87
CT of abdomen and pelvis with contrast material	3.57	156.78	9.9	507.38	7.26	372.23
CT of abdomen, pelvis, and kidney without contrast material	5.32	246.89	13.69	654.21	8.12	426.08

## Data Availability

Not applicable.
